# Physiological and Biochemical Responses of Pepper (*Capsicum annuum* L.) Seedlings to Nickel Toxicity

**DOI:** 10.3389/fpls.2022.950392

**Published:** 2022-07-18

**Authors:** Muhammad Ahsan Altaf, Yuanyuan Hao, Chengyao He, Muhammad Ali Mumtaz, Huangying Shu, Huizhen Fu, Zhiwei Wang

**Affiliations:** ^1^Key Laboratory for Quality Regulation of Tropical Horticultural Crops of Hainan Province, School of Horticulture, Hainan University, Haikou, China; ^2^Sanya Nanfan Research Institute of Hainan University, Hainan Yazhou Bay Seed Laboratory, Sanya, China

**Keywords:** nickel toxicity, heavy metal, pepper, metabolites, mineral homeostasis

## Abstract

Globally, heavy metal pollution of soil has remained a problem for food security and human health, having a significant impact on crop productivity. In agricultural environments, nickel (Ni) is becoming a hazardous element. The present study was performed to characterize the toxicity symptoms of Ni in pepper seedlings exposed to different concentrations of Ni. Four-week-old pepper seedlings were grown under hydroponic conditions using seven Ni concentrations (0, 10, 20, 30, 50, 75, and 100 mg L^–1^ NiCl_2_. 6H_2_O). The Ni toxicity showed symptoms, such as chlorosis of young leaves. Excess Ni reduced growth and biomass production, root morphology, gas exchange elements, pigment molecules, and photosystem function. The growth tolerance index (GTI) was reduced by 88-, 75-, 60-, 45-, 30-, and 19% in plants against 10, 20, 30, 50, 75, and 100 mg L^–1^ Ni, respectively. Higher Ni concentrations enhanced antioxidant enzyme activity, ROS accumulation, membrane integrity [malondialdehyde (MDA) and electrolyte leakage (EL)], and metabolites (proline, soluble sugars, total phenols, and flavonoids) in pepper leaves. Furthermore, increased Ni supply enhanced the Ni content in pepper’s leaves and roots, but declined nitrogen (N), potassium (K), and phosphorus (P) levels dramatically. The translocation of Ni from root to shoot increased from 0.339 to 0.715 after being treated with 10–100 mg L^–1^ Ni. The uptake of Ni in roots was reported to be higher than that in shoots. Generally, all Ni levels had a detrimental impact on enzyme activity and led to cell death in pepper seedlings. However, the present investigation revealed that Ni ≥ 30 mg L^–1^ lead to a deleterious impact on pepper seedlings. In the future, research is needed to further explore the mechanism and gene expression involved in cell death caused by Ni toxicity in pepper plants.

## Introduction

Rapid urbanization, industrialization, and modern agriculture have considerably escalated environmental degradation in developed countries, and resulted in an excessive accumulation of heavy metals in agricultural soils (Sardar et al., 2022a; Shahzadi et al., 2022). Recent studies have shown that contamination by heavy metals in agricultural soils has adverse effects on health and food security (Altaf M. M. et al., 2022). Because of the extensive availability of heavy metals in the environment, their residues enter and are consumed by plants. Plants readily absorb heavy metals, which further enter the food chain, causing major health risks to animals and humans (Heidari et al., 2020).

Ni is considered a common trace element among emerging pollutants. Ni is released into the environment from natural, as well as industry resources (Shahzad et al., 2018; Rahi et al., 2021). The release of Ni into the environment, especially its deposition in cultivated soils, is a major problem (Shukla et al., 2015). Its concentrations range from 10 to 40 ppm in most soils but exceed 1,000 ppm in serpentine soils or soils enriched with Ni-bearing ores (Klein et al., 2022). Nickel is a required nutrient for proper plant growth, however, excessive levels of Ni act as heavy metals, causing negative effects on plant growth, quality, and productivity (Rehman et al., 2022). Plant structural and anatomical dynamics are also influenced by excessive Ni concentration. In several agricultural crops, elevated Ni levels have been demonstrated to reduce plant height and biomass production (Seregin et al., 2003). In a wide range of plant species, excess Ni in plant growth medium disrupts physiological functions, generating detrimental effects on plant growth and toxicity signs, including chlorosis and necrosis (Valivand et al., 2019). Furthermore, Hermle et al. (2007) observed that Ni toxicity deforms chloroplast structures and reduces chlorophyll production. Higher Ni concentrations reduced leaf water potential, total moisture content, stomatal conductance, and transpiration rate in *Populus nigra* (Velikova et al., 2011). Elevated Ni levels in the soil disrupted plants’ several physiological processes, causing toxicity signs such as chlorosis and necrosis in *Solanum lycopersicum* (Jahan et al., 2020), *Brassica oleracea* (Shukla et al., 2015), and *Cucurbita pepo* (Valivand and Amooaghaie, 2021).

For appropriate growth and development of plants, optimal photosynthesis is a prerequisite. Excessive Ni exposure may cause non-specific photosynthetic limitation in plants, either directly or indirectly (Shahzad et al., 2018). Nickel stress may impair photosynthesis due to the reduction in activity of essential Calvin cycle enzymes. Furthermore, Alam et al. (2007) observed that photosynthesis-related enzymes might be targeted by Ni toxicity. Nickel stress considerably decreased leaf gas exchange elements, photosystem function, and osmotic balance in tomato (Jahan et al., 2020). Roots supply nutrients and water to plants’ aerial parts, and are the first organs to be affected by Ni (Altaf et al., 2021a). Recently, Jahan et al. (2020) observed that Ni toxicity dramatically reduces root growth and mineral nutrient homeostasis in tomato. Heavy metal remarkably reduces root growth traits in watermelon seedlings (Nawaz et al., 2018). In addition, Ni toxicity significantly reduces macro and micro-nutrient uptake, absorption, and translocation in zucchini seedlings, resulting in a nutritional shortage (Valivand et al., 2019). Under Ni toxicity, nutrient absorption declines as a result of disruptions in the structure and activity of enzymes related to cell membrane (Seregin et al., 2003).

Several studies have been performed to explore the deleterious impact of heavy metals (Shahzad et al., 2016). Such damaging effects are directly linked with the overproduction of ROS, which, through lipid peroxidation, negatively affects lipids, proteins, cell membranes, and DNA (Nawaz et al., 2018). Furthermore, Ni stress leads to the overproduction of ROS such as hydrogen peroxide (H_2_O_2_) and superoxide ion (O_2_^•–^) (Khaliq et al., 2015). MDA levels, which are a general biomarker of membrane degradation in redox homeostasis, and are increased dramatically in many plant species under exposure to high Ni concentrations (Kazemi et al., 2010). Jahan et al. (2020) noticed that ROS are signaling molecules, and plants subjected to Ni toxicity produce excessive amounts of ROS, as evidenced by greater levels of oxidative stress markers (H_2_O_2_ and O_2_^•–^). Plants have evolved diverse and effective management techniques to deal with metal-induced oxidative damage, and the antioxidative defense system is one of them (Jahan et al., 2020). Excessive Ni supply leads to overproduction of ROS, which ultimately results into oxidative damage to cauliflower (Shukla et al., 2015). Antioxidant enzymes ameliorate the deleterious effects of ROS by modulating their activity by altering gene expression under stressful environments (Chen et al., 2009). Moreover, Ni stress enhanced the antioxidant enzymes’ [Superoxide dismutase (SOD), catalase (CAT), ascorbate peroxidase (APX), and glutathione reductase (GR)] activity in tomato (Altaf et al., 2021a). Nickel stress markedly decreased SOD and APX activity in both the roots and leaves of pea plants, but increased the activity of glutathione *S*-transferase (GST) in leaves and roots (Gajewska and Skłodowska, 2005). Plants respond to Ni toxicity by activating antioxidant enzymes including SOD, CAT, APX, and GR; as well as producing non-enzymatic antioxidants such as glutathione, ascorbic acid, and proline, which may effectively eliminate ROS (Valivand et al., 2019). Plant natural compounds such as flavonoids, phenolics, and anthocyanins contribute to antioxidant capacity and stress tolerance. Zhang et al. (2016) reported that ROS levels are lowered and free radicals’ generation is limited with secondary metabolites.

*Capsicum* is a member of the Solanaceae family. It contains abundant quantities of vitamins A and C (Sarafi et al., 2017). Additionally, it contains nutrients such as potassium and phosphorus. Fresh, dried, or processed fruits are used as table vegetables or spices (Altaf et al., 2021b). Pepper is one of China’s most valuable commercial crops. China is the world’s largest producer, user, and exporter of pepper (Sarafi et al., 2017). Nickel toxicity has a deleterious impact on plant health in a wide range of plant species through disrupting antioxidant systems (Gajewska and Skłodowska, 2005; Jahan et al., 2020). Pepper’s physiological and biochemical reaction to nickel stress is still unclear. Main objectives of current study are: (1) to discuss the impact of excess Ni toward growth and metabolism of pepper seedlings cultivated under hydroponic conditions with high concentrations of Ni; (2) to assess the level of nickel toxicity in pepper leaves and roots. Additionally, the present study focused (3) to evaluate the influence of Ni toxicity on the photosynthetic machinery, and finally, (4) to understand the root growth pattern and mineral nutrient uptake under various Ni toxicity levels in pepper seedlings.

## Materials and Methods

### Experimental Material and Setup

In this experiment, pepper cultivar “Ca-59” (*Capsicum annuum* L.) was used to evaluate the toxicity level of Ni. Pepper seeds were collected from the vegetable seed bank of School of Horticulture, Hainan University, Haikou, Hainan, China. Nickel [Nickel chloride (NiCl_2_. 6H_2_O)] was bought from a Hainan Baihui Biotechnology Co., Ltd., Haikou, Hainan, China. The plant growth room is under climate-controlled conditions (Temperature 25°C ± 5; light/dark 16/8-h period; and relative humidity 60–85%). The pepper seeds were sown in 50-cell seedling trays filled with vermiculite media. After 30 days, the equal-sized seedlings were shifted into plastic containers of 4-liter capacity (6 plants per container), filled with Hoagland’s nutrient solution. By adding NaOH/HCl solution, the pH of the Hoagland nutrition solution was adjusted to 5.5 ± 1. The nutrition solution was replenished every fifth day to provide a constant supply of nutrients. After an adaptation period of 5 days, plants were separated into seven groups. Varying concentrations of Ni were applied to each group, viz. 0, 10, 20, 30, 50, 75, and 100 mg L^–1^. Plant samples were collected for further analysis after 14 days of Ni stress.

### Growth Traits and Root Morphology

After 14 days of Ni treatment, Shoots and roots were removed and weighed separately to determine fresh biomass, followed by placing the samples in an oven at 80°C for 3 days to determine dry weights. The growth tolerance index (GTI; in %) was calculated individually for roots and shoots using the dry weight procedure quoted by Altaf M. A. et al. (2022). Root harvesting was done by picking three uniform plants. Roots were washed with running tap water. Imagery scan screen (Epson Expression 11000XL, Regent Instruments, Canada) was used to perform root scanning. Further, WinRHIZO 2003a software (Regent Instruments) was used for root image analysis.

### Photosynthesis Related Parameters and Scanning Electron Microscopy

A portable photosynthesis system (CIRAS-3, Hansatech Co., United States) was used to measure gas exchange elements. Gas exchange parameters were calculated using fully developed leaves (Zhang et al., 2020). Lichtenhaler and Wellburn’s (1983) approach was used to analyze the pigment content of leaves. 80% acetone was used to crush and homogenized one gram of fresh plant material. 10 mL of this solution was centrifuged for 15 min at 3,000 rpm in test tubes. The absorption values were measured at 662, 645, and 470 nm. To measure chlorophyll fluorescence (CF), fully formed leaves were utilized, and leaf data was obtained between 9:00 and 11:00 a.m. using an IMAGING-PAM Chl fluorescence analyzer (Heinz Walz, Effeltrich, Germany) after 30 min of dark adaptation. The *F*_v_/*F*_m_ (maximum photochemical efficiency) value was determined in accordance with (Jahan et al., 2021).

To prepare leaf samples for SEM, they were rapidly treated first with glutaraldehyde (2.5%), followed by OsO4 (1%) in phosphate-buffered saline (0.1 M; PBS; pH 6.8) for avoiding any damage. A graded ethanol solution was used to dry the treated leaves, then shifted to an iso-amyl acetate + alcohol combination (1:1, v/v), and finally into iso-amyl acetate (used in pure form). Finally, samples were vacuum-dried with liquid CO_2_ in a Hitachi Model HCP-2 and coated with gold-palladium in a Hitachi Model E-1010 ion sputter. An S-4800 microscope (Hitachi Led., Tokyo, Japan, Model TM-1000) was used to record the SEM observations. For observing the contents of chlorophyll and carotenoid, TCS SP2 laser confocal microscope (Leica, Germany) was used, as previously reported by Mumtaz et al. (2022).

### Proline and Soluble Sugars

Bates et al. (1973) described a method for determining the proline content. A 0.5 g leaf sample was homogenized in 5 mL sulfosalicylic acid (3% m/v) and centrifuged at 12,000 *g* for 20 min at 4°C. The content of the proline was measured at 520 nm. To assess soluble sugars, the phenol-sulfuric acid approach was employed (Dubois et al., 1956). After homogenizing 0.1 g of dried leaves in deionized water, the extract was filtered and treated with 2% (w/v) phenol and 98% sulfuric acid. A spectrophotometer was used to measure the absorbance at 490 nm after 1 h of room temperature incubation.

### Secondary Metabolites

Leaf samples (0.2 g) were crushed using cold methanol (70% v/v) having formic acid (2% v/v) and ethanol (28% v/v), for measuring the secondary metabolites. The homogenized samples were first digested (30 min) and then stirred at 250 rpm for 2 h at 30°C. The samples were centrifuged at 10,000 *g* for 10 min at 4°C. The supernatant was then filtrated using a 0.45 M filter membrane for further analysis. According to Zhishen et al. (1999), the flavonoid content of leaves was measured. The total phenol content of leaves was determined using the method proposed by Singleton and Rossi (1965).

### Analysis of ROS, Malondialdehyde, and Antioxidant Enzymes

To determine the ROS (H_2_O_2_ and O_2_^•–^) level, MDA content, and antioxidant enzyme [SOD, CAT, APX, GR, GST, and peroxidase (POD)] activity, 0.5 g frozen tissue (leaf and root) samples were homogenized using liquid nitrogen. The ground tissue (leaf and root) samples were homogenized in 900 μL of 100 mM phosphate buffer (pH 7.4), as prescribed in the kit. Each homogenized sample was centrifuged at 12,000 × *g* for 15 min at 4°C. After that, the supernatant was added to a new falcon tube for further analysis. The activities of SOD, CAT, APX, GR, GST, and POD, and the levels of H_2_O_2_, O_2_^•–^, and MDA were calculated using the instructions included in kits (A001–1, A007–1, A123–1, A062–1, A004, A084-3-1, A064, A052, and A003-3, respectively) purchased from the Nanjing Jiancheng Bioengineering Institute in Nanjing, China. The absorbance values were measured at 550, 405, 290, 340, 412, and 420, 405, 550, and 530 nm, respectively.

### Histochemical Localization of O_2_^•–^ by Nitroblue Tetrazolium Staining, H_2_O_2_ by 3,3′ -Diaminobenzidine Staining, and Malondialdehyde by Schiff’s Reagent Staining

Nitroblue tetrazolium and DAB staining were used for histochemical staining of O_2_^•–^, and H_2_O_2_ as previously quoted by Altaf et al. (2021c). According to Pompella et al. (1987), freshly obtained leaves were stained in Schiff’s reagent for 60 min until a red color appeared, and then extra staining was removed by washing in potassium sulfite solution (0.5%, w/v, K_2_S_2_O_5_ in 0.05 M HCl).

### Ion Analysis

The method quoted by Altaf et al. (2021a) was used to determine the Ni content, samples of roots and leaves were harvested individually. Oven-dried samples were ground and digested at 80°C with HNO_3_:HClO_4_ (5:1 v/v). The concentration of Ni in leaves and roots was determined using an atomic absorption spectrophotometer (Z-5000, Hitachi, Japan). The procedure described by Altaf et al. (2021c) was used to determine the nutritional element content of pepper seedling roots and leaves. After harvesting, samples of leaves and roots were obtained and washed multiple times with ultrapure water before being oven-dried at a temperature of 65°C. Following that, dried samples (leaves and roots) were digested separately in 3 mL of 1 M HNO_3_, then samples were boiled for 10 min at 95°C. Finally, N, P, and K contents were measured using an inductively coupled plasma optical emission spectrometer (ICP-OES; SPS3100, SII Nano Technology, Japan).

### Statistical Analysis

The statistical analysis was carried out using statistics 10.1 software. Different letters indicate a significant difference in Ni concentrations (*p* ≤ 0.05). The values in the figures are always expressed as the mean standard error of four independent replicates. Fisher’s least significant difference (LSD) (*p* ≤ 0.05) test was used to determine the differences in Ni concentrations (*p* 0.05).

## Results

### Plant Growth and Growth Tolerance Index

**FIGURE 1:**
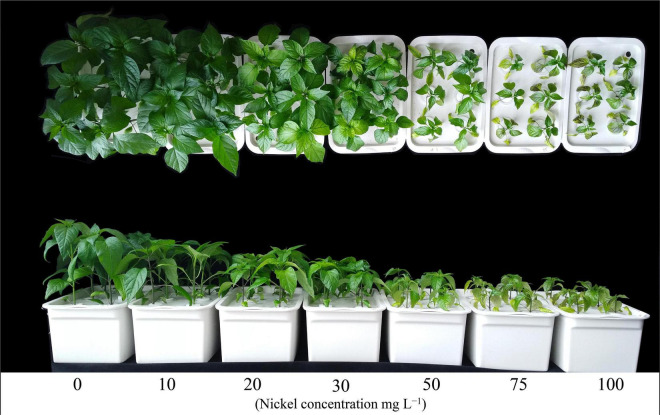
Performance of pepper seedlings at various nickel concentrations.

Various Ni concentrations (10–100 mg L^–1^) dramatically reduced the growth of pepper seedlings (Figure 1). Additionally, we observed that increasing the Ni concentration reduced root growth and development. Nickel concentrations considerably decreased the root and shoot biomass (fresh and dry) of pepper seedlings, but the decline was more pronounced at higher concentrations (50, 75, and 100 mg L^–1^ Ni) than at lower concentrations (10, 20, and 30 mg L^–1^ Ni). The fresh shoot weight and dry shoot weight were dramatically reduced by 10. 43-, 22. 03-, 32. 47-, 48. 76-, 62. 48-, and 73.03% and 8. 92-, 19. 22-, 32. 14-, 50. 35-, 68. 28-, and 79.64%, respectively, following treatment with 10, 20, 30, 50, 75, and 50 mg L^–1^ Ni, respectively, when compared with the CK-seedlings (Table 1). Similarly, fresh root weight and dry root weight were decreased by 12. 22-, 25. 18-, 38. 14-, 55. 18-, 69. 88-, and 80.01% and 10. 25-, 24. 17-, 39. 56-, 53. 47-, 68. 49-, and 81.31%, respectively, when compared to CK plants (Table 1). By increasing the Ni concentrations, the GTI was continuously decreasing, indicating a negative correlation between the Ni concentrations and the GTI. The GTI_S_ was 87-, 75-, 61-, 44-, 30-, and 20%, and GTI_R_ was 89-, 75-, 60-, 46-, 31-, and 18% in pepper seedlings against 10, 20, 30, 50, 75, and 100 mg L^–1^ Ni, respectively (Table 1).

**TABLE 1:** Effect of nickel on fresh and dry biomass of pepper seedlings.

Nickel (mg L^–1^)	Biomass yield per plant (g)				Growth tolerance Index (%)	
	Fresh		Dry		Shoot	Root
	Shoot	Root	Shoot	Root		
0	8.56 ± 0.23a	2.88 ± 0.063a	2.70 ± 0.066a	0.273 ± 0.007a	100	100
10	7.64 ± 0.20b	2.55 ± 0.078b	2.37 ± 0.063b	0.240 ± 0.004b	87	89
20	6.65 ± 0.22c	2.26 ± 0.058c	2.02 ± 0.066c	0.207 ± 0.007c	74	75
30	5.76 ± 0.17d	1.90 ± 0.063d	1.67 ± 0.072d	0.165 ± 0.007d	61	60
50	4.37 ± 0.15e	1.39 ± 0.066e	1.21 ± 0.075e	0.121 ± 0.006e	44	46
75	3.21 ± 0.16f	0.86 ± 0.049f	0.81 ± 0.057f	0.082 ± 0.005f	30	31
100	2.30 ± 0.16g	0.57 ± 0.061g	0.54 ± 0.049g	0.051 ± 0.004g	20	18

*Mean ± SE of four replicates. The lowercase letters in each column demonstrate a statistically significant difference from one another according to the fisher LSD test at P ≤ 0.05.*

### Root Morphology

**FIGURE 2:**
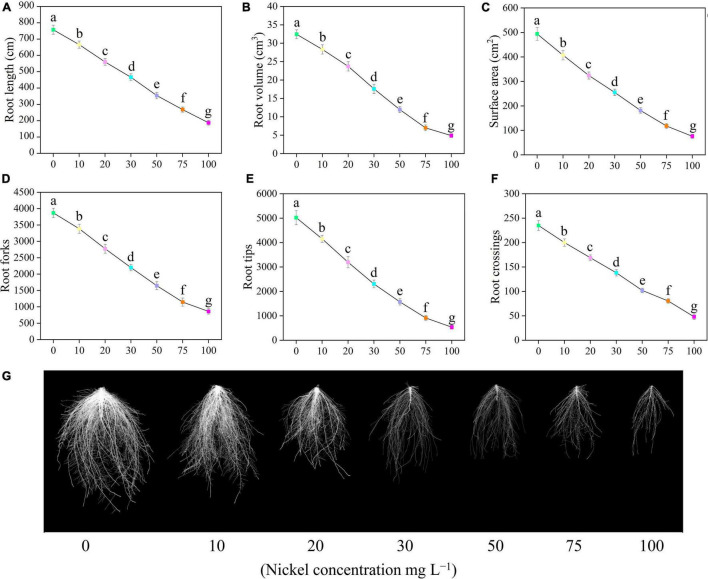
Effect of nickel on root morphology in pepper seedlings. Means ± SE, *n* = 4, the lowercase letters demonstrate a statistically significant difference from one another according to the fisher LSD test at *P* ≤ 0.05.

Root growth of pepper seedlings was significantly reduced by Ni concentrations, as shown by a reduction in total root length, volume, surface area, tips, forks, and crossings (Figure 2). Furthermore, when the Ni concentration was increased, the root morphology-related traits were progressively decreased, but the most harmful impact was seen at 50, 75, and 100 mg L^–1^ Ni when compared to CK plants (Figure 2). The root length, root volume, and surface area, were decreased by 12. 02-, 26. 21-, 38. 44-, 53. 23-, 64. 59-, and 75.29% (Figure 2A); 12. 70-, 26. 91-, 45. 76-, 63. 12-, 78. 44-, and 84.92% (Figure 2B); and 17. 61-, 34. 41-, 48. 38-, 63. 36-, 76. 11-, and 84.61% (Figure 2C); after being treated with 10, 20, 30, 50, 75, and 100 Ni mg L^–1^, respectively, when compared to untreated plants (CK-group). Furthermore, significant changes were noticed after 20–100 mg L^–1^ Ni, and showed reduced root forks from 12.48 to 77.76% (Figure 2D), root tips from 17.30 to 89.34% (Figure 2E), and root crossings from 14.89 to 79.57% (Figure 2F).

### Leaf Gas Exchange Elements

**FIGURE 3:**
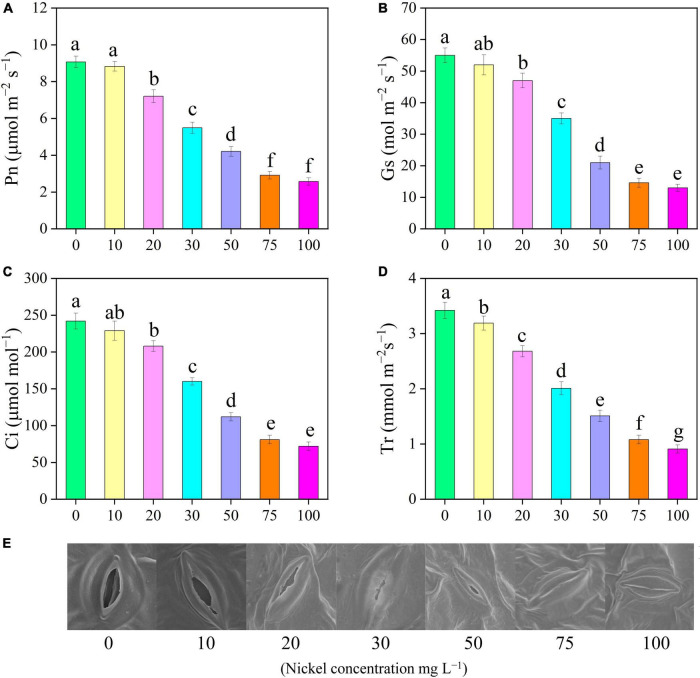
Effect of nickel on leaf gas exchange parameters and stomatal characteristics in pepper seedlings. Means ± SE, *n* = 4, the lowercase letters demonstrate a statistically significant difference from one another according to the fisher LSD test at *P* ≤ 0.05.

Our findings clearly demonstrated that all Ni concentrations caused significant effect on the photosynthetic activities of pepper seedlings (Figures 1, 3). The Pn rate of pepper seedlings receiving 10 mg L^–1^ Ni was observed to be similar to that of the CK group (Figure 3A). In addition, compared with CK, the photosynthetic assimilation (Pn) was decreased by 39. 47-, 53. 58-, 67. 91-, and 71.66%, and stomatal conductance (Gs) rate was decreased by 36. 36-, 61. 81-, 73. 45-, and 76.36%, respectively, when pepper seedlings were subjected to 30, 50, 75, and 100 mg L^–1^ Ni respectively (Figures 3A,B). The pepper plants treated with 10 and 20 mg L^–1^ Ni showed statistically similar results in Pn and Gs (Figures 3A,B). Furthermore, the intercellular CO_2_ (Ci) and transpiration rate (Tr) were considerably decreased by 14. 04-, 33. 88-, 53. 71-, 66. 52-, and 70.24%, and 21. 63-, 41. 22-, 55. 84-, 68. 42-, and 73.39%, respectively, following treatment with 20, 30, 50, 75, and 100 mg L^–1^ Ni (Figures 3C,D).

### Stomatal Characteristics of Pepper Leaves

Scanning electron microscopic analysis showed that various Ni concentrations have a significant effect on stomatal morphology. SEM revealed that stomatal length and width were smaller in the leaves of pepper subjected to different Ni concentrations than those of the control leaves. Figure 3E shows that when the Ni concentration increased, the stomatal opening degree gradually decreased. Under higher Ni concentrations (75, and 100 mg L^–1^ Ni), the stomata of pepper were damaged and subsided (Figure 3E).

### Chlorophyll Content and SPAD Index

**FIGURE 4:**
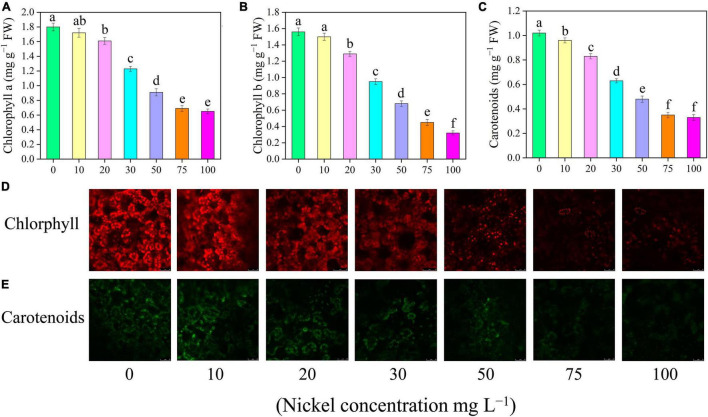
Effect of nickel on photosynthetic pigments, carotenoids, and auto-fluorescence microscopic observations in pepper seedlings. Means ± SE, *n* = 4, the lowercase letters demonstrate a statistically significant difference from one another according to the fisher LSD test at *P* ≤ 0.05.

The findings showed that increasing Ni concentrations from 0 to 100 mg L^–1^ substantially lowered photosynthetic pigments (Figures 4A–E). The addition of 20, 30, 50, 75, and 100 mg L^–1^ Ni to pepper plants reduced the chlorophyll a and chlorophyll b by 10. 55-, 31. 66-, 49. 44-, 61. 66-, and 63.88% and 17. 30-, 39. 10-, 56. 41-, 71. 15-, and 79.48%, respectively (Figures 4A,B). Additionally, the amounts of pigment molecules (chlorophyll a and b) in the leaves of pepper seedlings that were treated with 0 and 10 mg L^–1^ Ni were the same (Figures 4A,B,D). Carotenoids in pepper leaves were reduced by 5. 88-, 18. 62-, 38. 23-, 52. 94-, 65. 68-, and 67.64%, when treated with 10, 20, 30, 50, 75, and 100 mg L^–1^ Ni, respectively, compared to CK seedlings (Figures 4C,E).

### Chlorophyll Fluorescence Traits

**FIGURE 5:**
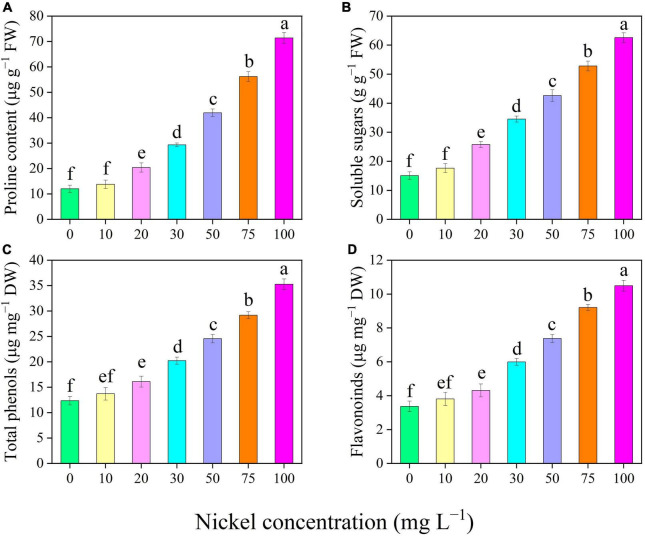
Effect of nickel on chlorophyll fluorescence parameters in pepper seedlings. Means ± SE, *n* = 4, the lowercase letters demonstrate a statistically significant difference from one another according to the fisher LSD test at *P* ≤ 0.05.

Nickel concentrations caused considerable alterations in pepper seedling CF characteristics (Figure 5). The maximum quantum yield of photosystem II (*F*_v_/*F*_m_), effective quantum efficiencies of the PSII [Y(II)], non-photochemical quenching (NPQ), and photochemical quenching coefficient (qP) of pepper leaves were evaluated, to describe how photosystem II functions work under different Ni concentrations (Figure 5). In addition, when the Ni concentration was increased, the *F*_v_/*F*_m_, Y(II), and qP were reduced, but the NPQ value was enhanced (Figure 5). In addition, compared to CK, the *F*_v_/*F*_m_ was reduced by 12. 02-, 28. 20-, 42. 08-, 54. 79-, and 62.65%, Y(II) was decreased by 18. 50-, 35. 62-, 52. 25-, 66. 50-, and 71.25%, and qP was reduced by 15. 77-, 29. 78-, 44. 97-, 66. 01-, and 68.34%, when plants were exposed to 20, 30, 50, 75, and 100 mg L^–1^ Ni, respectively (Figures 5A–C). Furthermore, the NPQ values were significantly increased by 0. 47-, 1. 02-, 1. 51-, 1. 95-, and 2.26-fold, respectively, following treatment with 20, 30, 50, 75, and 100 mg L^–1^ Ni, respectively (Figure 5D).

### Osmolytes and Secondary Metabolites

**FIGURE 6:**
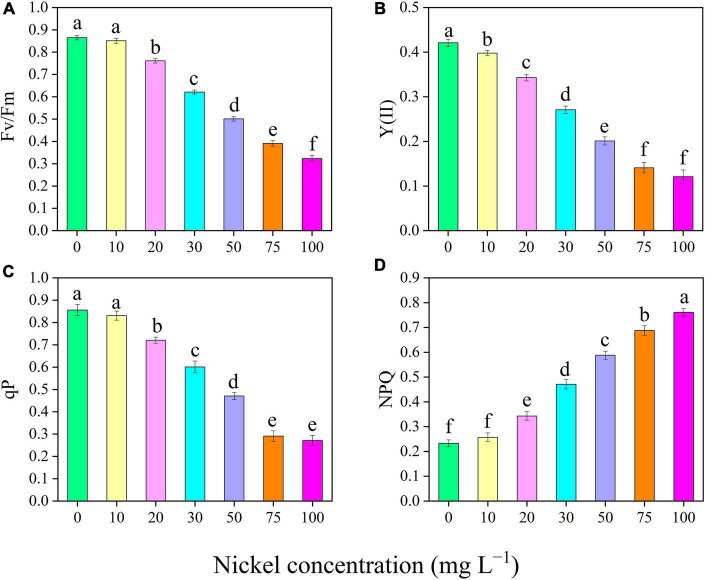
Effect of nickel on osmolytes and secondary metabolites traits in pepper seedlings. Means ± SE, *n* = 4, the lowercase letters demonstrate a statistically significant difference from one another according to the fisher LSD test at *P* ≤ 0.05.

There was a significant increase in the proline and soluble sugar content of the leaves when Ni was supplied (Figures 6A,B). The proline and soluble sugar content were efficiently improved up to 0. 69-, 1. 43-, 2. 48-, 3. 67-, and 4.93-fold and 0. 71-, 1. 29-, 1. 83-, 2. 50-, and 3.15-fold, respectively, following treatment with 20, 30, 50, 75, and 50 mg L^–1^ Ni, respectively, when compared with the CK-group (Figures 6A,B). Similarly, when pepper seedlings were exposed to 20, 30, 50, 75, and 100 mg L^–1^ Ni, the total phenolics and flavonoids content were increased by 0. 31-, 0. 63-, 0. 98-, 1. 36-, and 1.85-fold and 0. 27-, 0. 77-, 1. 18-, 1. 73-, and 2.11-fold, respectively, when compared to CK plants (Figures 6C,D). The total phenolics and flavonoid content in the pepper leaves were statistically similar in the treatments of 0, 10, and 20 mg L^–1^ Ni (Figures 6C,D).

### Oxidative Damage

**FIGURE 7:**
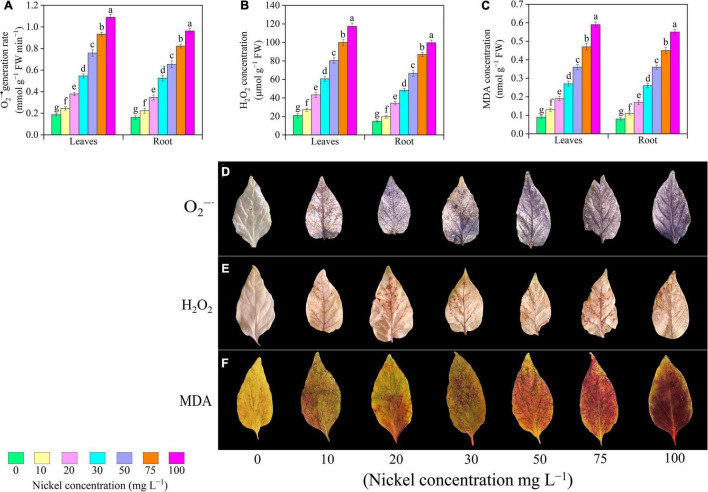
Effect of nickel on oxidative stress biomarkers in pepper seedlings and histochemical localization of O_2_^–^ **(D)** H_2_O_2_ **(E)**, and MDA **(F)** accumulation. Means ± SE, *n* = 4, the lowercase letters demonstrate a statistically significant difference from one another according to the fisher LSD test at *P* ≤ 0.05.

The pepper seedlings were exposed to different Ni concentrations, the H_2_O_2_ and O_2_^•–^, and MDA levels of pepper leaves were enhanced when compared to CK plant (Figure 7). When pepper plants were subjected to 10, 20, 30, 50, 75, and 100 mg L^–1^ Ni, the O_2_^•–^ generation rate in leaves/roots was increased 0.31/0. 37-, 1.03/1. 12-, 1.93/2. 24-, 3.06/3. 03-, 4.01/4. 08-, and 4.84/4.94-fold, respectively, when compared to CK seedlings (Figure 7A). H_2_O_2_ concentration in pepper leaves/roots was improved 0.29/0. 34-, 1.06/1. 34-, 1.88/2. 29-, 2.82/3. 54-, 3.75/4. 92-, and 4.58/5.79-fold, respectively, following treatment of 10, 20, 30, 50, 75, and 100 mg L^–1^ Ni compared with CK-group (Figure 7B). Furthermore, Ni application considerably increased the MDA level of pepper seedlings. Additionally, a significant increase was observed after 10–100 mg L^–1^ Ni, and showed enhancement in MDA leaves/roots from 0.55/0.48- to 3.91/3.21 (Figure 7C). Under histochemical staining, dark blue dots indicate O_2_^•–^, while brown spots represent H_2_O_2_ accumulation in pepper leaves, as shown in Figure 7D. After 2 weeks of Ni-treatment, we noticed that by increasing Ni concentration, a greater accumulation of O_2_^•–^ can be seen in Ni-stressed leaves (Figure 7D). Similarly, a considerable increase in H_2_O_2_ and MDA accumulation can be seen (Figures 7E,F).

### Antioxidant Enzymes

**FIGURE 8:**
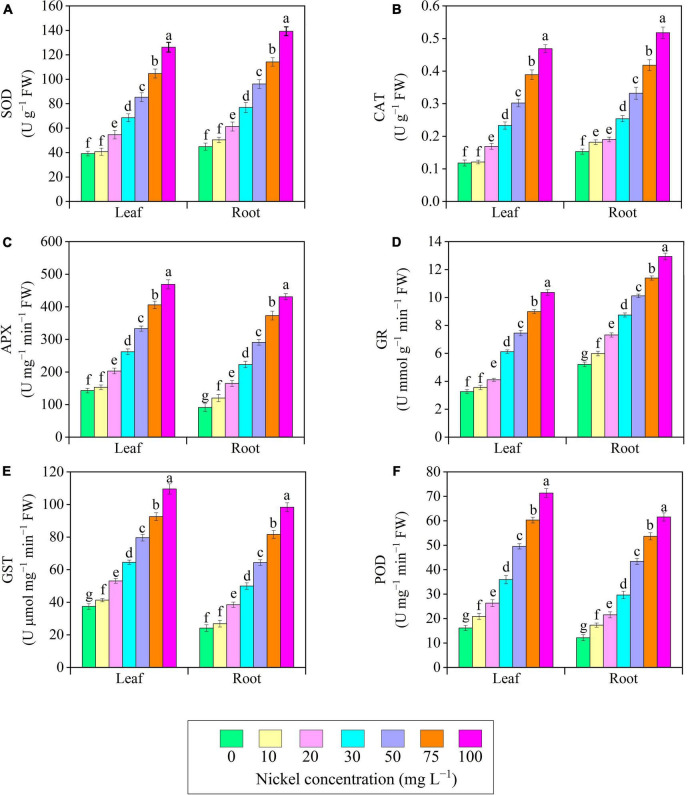
Effect of nickel on antioxidant enzymes activity in pepper seedlings. Means ± SE, *n* = 4, the lowercase letters demonstrate a statistically significant difference from one another according to the fisher LSD test at *P* ≤ 0.05.

There were significant changes in antioxidant enzyme activities when plants were exposed to different Ni doses; a positive relationship was observed between Ni supplementation and antioxidant enzyme activities (Figure 8). The present results revealed that in the leaves’ activity of SOD, CAT, APX, and GR remained similar at 10 mg L^–1^ Ni when compared to CK-group (Figures 8A–D). The activities of antioxidant enzymes (SOD, CAT, APX, GR, GST, and POD) in leaves/roots were improved consistently as Ni level increased from 20 to 100 mg L^–1^, with the maximum increase in activity of antioxidant enzyme occurring at 75 and 100 mg L^–1^ Ni. Additionally, a significant increase was noticed after 20–100 mg L^–1^ Ni, and showed improvement in SOD leaves/roots from 0.39/0.39- to 2.22/2.09-fold (Figure 8A), CAT leaves/roots from 0.43/0.24- to 2.97/2.38-fold (Figure 8B), APX leaves/roots from 0.41/0.81- to 2.27/3.73-fold (Figure 8C), GR leaves/roots from 0.26/0.41- to 2.17/1.48-fold (Figure 8D), GST leaves/roots from 0.41/0.59- to 1.92/3.06-fold (Figure 8E), and POD leaves and roots from 0.63/1.77 to 3.42/5.06-fold (Figure 8F).

### Mineral Nutrient Uptake

The macronutrient (Nitrogen, N; Phosphorus, P; and Potassium, K) uptake in pepper (leaves and roots) was examined to expose the influence of Ni on nutrient homeostasis, as shown in Table 2. Ni-supplementation effectively reduced the N, P, and K concentration of leaves and roots (Table 2). The N, P, and K uptake were decreased 9.91/6. 99-, 21.50/20. 69-, 36.02/35. 49-, 47.19/51. 41-, 61.79/60. 21-, and 67.98/64.58%, (Table 2); 10.54/7. 84-, 24.72/17. 21-, 37.47/29. 20-, 54.72/41. 58-, 66.92/55. 11-, and 73.07/63.48% (Table 2); and 10.19/8. 21-, 21.40/19. 86-, 31.97/34. 35-, 42.98/50. 28-, 56.11/63. 72-, and 66.74/75.21% (Table 2), in the leaves/roots of pepper seedlings, after being treated with 10, 20, 30, 50, 75, and 100 mg L^–1^ Ni, respectively, when compared to CK- group.

**TABLE 2:** Effects of nickel on macronutrient (Nitrogen, phosphorus, and potassium), uptake (in roots and shoot) in pepper seedlings.

Nickel (mg L^–1^)	Leaves (g kg^–1^ DW)			Root (g kg^–1^ DW)		
	Nitrogen	Phosphorus	Potassium	Nitrogen	Phosphorus	Potassium
0	41.72 ± 0.76 a	9.10 ± 0.22 a	58.39 ± 1.15 a	48.51 ± 1.14 a	13.1 ± 0.16 a	49.34 ± 0.69 a
10	37.22 ± 0.71 b	8.14 ± 0.14 b	52.44 ± 1.36 b	44.77 ± 1.21 b	11.9 ± 0.10 b	45.29 ± 0.76 b
20	32.89 ± 1.14 c	6.85 ± 0.15 c	45.89 ± 1.16 c	38.59 ± 1.45 c	10.7 ± 0.15 c	39.54 ± 0.97 c
30	26.71 ± 1.02 d	5.69 ± 0.20 d	39.72 ± 0.90 d	31.32 ± 1.18 d	9.21 ± 0.11 d	32.39 ± 1.27 d
50	22.02 ± 0.94 e	4.12 ± 0.12 e	33.29 ± 1.22 e	23.62 ± 1.09 e	7.61 ± 0.23 e	24.53 ± 1.28 e
75	15.91 ± 0.70 f	3.01 ± 0.11 f	25.63 ± 1.14 f	18.82 ± 0.93 f	5.84 ± 0.16 f	17.91 ± 0.85 f
100	13.44 ± 0.45 g	2.45 ± 0.14 g	19.42 ± 1.40 g	15.66 ± 0.85 g	4.75 ± 0.17 g	12.23 ± 1.24 g

*Mean ± SE of four replicates. The lowercase letters in each column demonstrate a statistically significant difference from one another according to the fisher LSD test at P ≤ 0.05.*

### Plant Tissues Nickel Concentration, Uptake, and Root to Shoot Translocation

The results indicated that when the Ni level increased, the Ni concentration in the tissues (root and shoot) improved significantly. The Ni supplementation showed a significant accumulation in pepper roots and shoots as compared to the CK-group (Table 3). When seedlings were treated with 50, 75, and 100 mg L^–1^ Ni, the highest Ni accumulation was 403. 3-, 499. 6-, and 585.6 mg kg^–1^ DW in the root and 237. 6-, 325. 3-, and 419.1 mg kg^–1^ DW in the shoots, respectively, when compared to the CK plants (Table 2). The findings indicate that Ni was accumulating more in the roots than in the shoots. Nickel absorption by shoots and roots increased considerably with increasing Ni levels. Additionally, increasing Ni concentration enhanced root-to-shoot Ni translocation (Table 3).

**TABLE 3:** Effects of nickel-on-nickel translocation (root to shoot), nickel concentrations and uptake (in roots and shoot; mg kg^–1^ DW) in pepper seedlings.

Nickel (mg L^–1^)	Ni concentration		Ni uptake		Ni translocation (Root to shoot)
	Root	Shoot	Root	Shoot	
0	22.33 ± 1.45 g	7.311 ± 0.95 g	6.069 ± 0.23 e	21.22 ± 2.52 f	0.327 ± 0.036 f
10	103.3 ± 5.04 f	35.01 ± 3.46 f	25.41 ± 1.86 d	89.69 ± 10.4 e	0.339 ± 0.033 e
20	196.1 ± 8.95 e	81.67 ± 6.35 e	40.76 ± 3.20 c	185.8 ± 19.1 d	0.414 ± 0.013 d
30	286.1 ± 8.14 d	149.3 ± 5.78 d	47.31 ± 3.55 ab	284.9 ± 20.4 b	0.521 ± 0.005 c
50	403.3 ± 9.59 c	237.6 ± 7.83 c	51.23 ± 4.35 a	331.7 ± 24.5 a	0.589 ± 0.014 bc
75	499.6 ± 11.4 b	325.3 ± 6.64 b	42.88 ± 2.42 bc	281.2 ± 20.1 bc	0.652 ± 0.024 ab
100	585.6 ± 8.81 a	419.1 ± 11.5 a	30.16 ± 3.82 d	243.1 ± 32.4 c	0.715 ± 0.009 a

*Mean ± SE of four replicates. The lowercase letters in each column demonstrate a statistically significant difference from one another according to the fisher LSD test at P ≤ 0.05.*

## Discussion

In recent years, agricultural soils have been polluted by a wide variety of environmental contaminants emerging from a wide range of sources. Heavy metals including Ni, cadmium, vanadium, and chromium are significant cause of agricultural soil contamination. Nickel has become a hazardous element. Nickel pollution of the environment, notably agricultural soils, is a major problem. The excessive Ni environmental deposition (including agricultural lands, water and atmosphere) caused by increased industrial use, has garnered recent attention (Shahzad et al., 2018). Over the last several decades, Ni has gained considerable attention due to its excessive industrial use and deposition in the environment. Thus, important processes and mechanisms implicated in Ni toxicity must be examined.

Plant growth characteristics, including shoot length, leaf area, size, and biomass production, are often regarded as the best indicators of any kind of metal toxicity and are also used to determine a plant’s tolerance capacity for metal toxicity. Heavy metals caused toxicity and had a detrimental effect on plant growth elements such as plant height and fresh and dry plant weights, and this kind of decrease often varies from organ to organ of the plants (Altaf et al., 2020). In this study, we observed that excessive nickel toxicity caused a significant decline in the fresh and dry weight of the roots and shoots of pepper seedlings (Table 1). The present results revealed a negative relationship between seedling growth traits and Ni concentration. Similarly, Shukla et al. (2015) stated that excessive Ni (0.5 mM) toxicity remarkably decreased biomass production, leaf area, and size of the cauliflower. Nickel supplementation considerably reduced the growth yield and quality of cucumber (Tabatabaei, 2009). Furthermore, Ni (50 μM) toxicity effectively reduced the growth of tomato seedlings by damaging the root architecture system and photosynthetic apparatus (Jahan et al., 2020). Seedling growth of *Cucurbita pepo*, *Calendula tripterocarpa*, *Solanum melongena*, and *Allium cepa* was inhibited by higher Ni concentrations (Alibakhshi and Khoshgoftarmanesh, 2015; Heidari et al., 2020; Shah et al., 2021; Valivand and Amooaghaie, 2021). Higher Ni levels interrupt normal developmental processes by causing a nutritional imbalance and disrupting their function in anabolic pathways.

Root size and root structure are important characteristics that influence the efficiency of nutrient absorption in plants. Contamination by heavy metals may induce alterations in root architecture (Altaf M. A. et al., 2022). The present results revealed that higher Ni levels dramatically hamper the root traits such as root length, volume, projected area, average diameter, surface area, tips, crossing, and forks (Figure 2). Similarly, Jahan et al. (2020) reported that Ni toxicity damages the root architecture system of tomato. Nickel accumulation in roots may induce nutritional distraction by disrupting root morphology. In maize, the accumulation of Ni in the pericycle hampered root branching. Nickel hindered root formation, which resulted in reduced cell division (Seregin et al., 2003). Pandey and Gopal (2010) observed that Ni (400 μM) stress influences the root growth, biomass production, and metabolism of eggplant. Mitotic cell division may be restricting root tip formation, resulting in a reduction in root length (Meisch et al., 1977). These findings are consistent with those previously published by Kabala and Janicka-Russak (2011) in cucumber, Gajewska and Skłodowska (2005) in pea, Page and Feller (2005) in wheat, and Li et al. (2009) in barely under Ni toxicity.

Photosynthesis is an important physiological mechanism in plants that regulates crop productivity and survivability. Chlorophyll is the primary energy source for photosynthesis (Yu-chen et al., 2015). Plants exposed to high Ni, particularly directly or indirectly, may induce non-specifications in the photosynthetic process (Krupa et al., 1993). In this study, excessive Ni concentration effectively decreased gas exchange characteristics, including Pn, Gs, Ci, and Tr (Figure 3). Alam et al. (2007) observed that Ni toxicity decreased chlorophyll content and net photosynthetic rate of *Brassica juncea*. In another study, Nawaz et al. (2018) and Jahan et al. (2020) revealed that heavy metals (Ni and Vanadium) toxicity significantly reduced gas exchange elements in watermelon and tomato. The outcomes of this research are similar with those of prior studies that measured photosynthesis in response to various heavy metal stresses, e.g., tomato under iron toxicity (Ahammed et al., 2020), cucumber and *Vinca rosea* under Ni toxicity (Tabatabaei, 2009; Khan et al., 2017) and pepper under vanadium and boron toxicity (Sarafi et al., 2017; Altaf et al., 2021b). Plants produce a wide range of pigment compounds. Primary pigment molecules for photosynthesis include chlorophyll a, chlorophyll b, and carotenoids. Nickel toxicity considerably reduced pigments content (Chlorophyll a, chlorophyll b and carotenoids) in pepper seedling (Figures 4A–C). Shukla et al. (2015) observed that high Ni concentration remarkably decreased photosynthetic pigment content in the leaves of cauliflower. Heavy metal (Ni, boron, and cadmium) application effectively hindered pigment levels in pepper, cucumber, and tomato leaves (Sarafi et al., 2017; Shah et al., 2019; Jahan et al., 2020). Photosynthesis pigments were decreased during V stress, which might be a result of oxidative stress-induced alterations in membrane permeability and component degradation (Aihemaiti et al., 2019). The formation of ROS is an important factor in the reduction of leaf chlorophyll concentration (Chary et al., 2008). Similar results were showed in watermelon (Nawaz et al., 2018), fenugreeks (Choudhary et al., 2012), cabbage (Shah et al., 2020a) tomato (Hasan et al., 2015), and in cucumber (Cao et al., 2019) under metals toxicity. Generally, increased chlorophyll content results in increased photosynthesis and hence improved plant performance. This is consistent with the findings of Wu et al. (2021) in strawberry under cadmium toxicity. CF has become a strong tool for studying plant photosynthetic attributes under abiotic stress. Nickel-induced photosynthesis inhibition, which results in a breakdown of the photosynthetic machinery and a decrease in the optimum *F*_v_/*F*_m_, is regarded as an efficient regulator of photosystem II for achieving a photo-oxidative output (Wang et al., 2013). Jahan et al. (2020) reported that Ni toxicity causes photoinhibition in the PSII reaction center. In line with these results, we observed that Ni toxicity effectively reduced *F*_v_/*F*_m_, PSII, and qP (Figure 5). The decrease in *F*_v_/*F*_m_, PSII, and qP, as well as the increase in NPQ, revealed that Ni stress caused serious damage to the photosynthetic machinery in pepper seedlings. These findings were consistent with prior studies on *Solanum lycopersicum*, *Cucumis sativus*, and *Zea maize* (Zhang et al., 2013; Martinez et al., 2018; Huang et al., 2019).

The most important metabolites synthesized in plant tissues under stressful situations is proline and soluble sugars. Proline and soluble sugar accumulation were associated with decreased leaf water content, ultimately leading to cellular dehydration and osmotic stress in pepper seedlings (Figures 6A,B). Jahan et al. (2020) supported these findings, suggesting that tomato seedlings under Ni toxicity demonstrate reciprocal behavior by producing proline in response to increasing cellular dehydration. Proline and soluble sugar levels were observed to be increased in stressful situations (Okunlola et al., 2018). Furthermore, the levels of proline and soluble sugar were enhanced in *Eruca sativa* (Kamran et al., 2016, *Triticum aestivum* (Gajewska et al., 2006), *Catharanthus roseus* (Yasin et al., 2018), and *Cucurbita pepo* (Valivand et al., 2019) under Ni toxicity. Phenols and flavonoids are secondary metabolites with antioxidant properties that serve as a second line of defense against free radical scavenging. In Pepper seedlings exposed to Ni-stress, the level of phenols and flavonoids in their leaves increased compared to CK-treatment (Figures 8C,D). These results agreed with previous research on pepper and rosemary under metal (boron and arsenic) toxicity (Sarafi et al., 2017; Farouk and Al-Amri, 2019). In addition, Ni stress enhanced the content of flavonoids in *Hibiscus sabdariffa* (Aziz et al., 2007).

Several investigations into the deleterious effects of heavy metals on biological membranes and integrity have been addressed. Under stressful environment, plants normally generate a large amount of ROS, which results in membrane lipid peroxidation and oxidative damage (Huang et al., 2019). When seedlings interact with heavy metals, they produce large amounts of H_2_O_2_ and O_2_^•–^, which is a primary component of ROS. The present results revealed that excessive Ni concentration significantly enhanced the level of H_2_O_2_ and O_2_^•–^ in the leaves and roots of pepper seedlings (Figures 7A,B). Similarly, Valivand et al. (2019) observed that Ni application effectively increased the overproduction of ROS in zucchini seedlings. In addition, several researchers supported these results, under heavy metal (Ni, vanadium, copper, cadmium, and boron) stress, the levels H_2_O_2_ and O_2_^•–^ were improved in watermelon, cucumber, tomato, and pepper seedlings (Sarafi et al., 2017; Nawaz et al., 2018; Cao et al., 2019; Jahan et al., 2020; Shah et al., 2020b). MDA is a significant indicator of oxidative damage to the integrity of cell membranes. In this study, Ni supplementation considerably increased MDA levels in the leaves and roots of pepper seedlings (Figures 7C,D). In a recent study, Altaf et al. (2021a) found that Ni application significantly enhanced the level of MDA in tomato seedlings. Furthermore, the results of our study exhibited a reduction in oxidative damage under Ni toxicity, which is also affirmed by extended literature on cucumber (Zhang et al., 2013), gladiolus (Zaheer et al., 2018), strawberry (Wu et al., 2021), and pea (Gajewska and Skłodowska, 2005) under metal toxicity.

Under abiotic stress conditions, the antioxidative defense system and redox balance in plants play a key role in limiting ROS production and mitigating oxidative stress. In this study, we found that antioxidant enzymes (SOD, CAT, APX, GR, GST, and POD) activity were improved with excessive Ni concentration (Figure 8). Similarly, when pea seedlings were exposed to Ni toxicity, the activity of antioxidant (SOD, GR, and POD) enzymes was improved (Rao and Sresty, 2000). Heavy metal ions may have both direct and indirect impacts on the formation of free oxygen radicals, resulting in an increase in enzymatic activity (Zembala et al., 2010). Ahammed et al. (2020) reported that under iron toxicity antioxidant enzymes activity were enhanced in *C. sativus*. The results of this study are comparable with those of Sarafi et al. (2017) in pepper seedlings exposed to boron toxicity, Choudhary et al. (2012) in fenugreeks subjected to cadmium toxicity, Altaf et al. (2021a) in tomato under Ni toxicity, in soybean exposed to Ni toxicity (Einhardt et al., 2022), and Valivand et al. (2019) in zucchini seedlings exposed to Ni toxicity. Antioxidant enzymes scavenge ROS and other free radicals, protecting plant cell membranes from damage.

Heavy metal toxicity impairs mineral ion uptake in plant roots (Li et al., 2021). In this study, Ni toxicity dramatically reduced the uptake of macronutrients (Table 2). Similarly, Yang et al. (1996) reported that Ni toxicity effectively decreased mineral nutrient flux in cabbage and clover. In a recent study, Valivand and Amooaghaie (2021) found that under nickel toxicity, the uptake of N, P, and K in the leaves and roots considerably declined in *C. pepo*. Furthermore, Ni absorption and accumulation in pepper shoots and roots increased as Ni concentrations increased. The root has a higher Ni concentration than the shoot tissues (Table 3). In tomato, Jahan et al. (2020) noticed that roots absorb a greater amount of Ni than the leaves. In addition, the concentration of vanadium was higher in tomato roots than in leaves (Altaf et al., 2021c). The present study revealed that Ni accumulation in pepper seedlings not only interferes with mineral nutrient intake, but also impairs nutrient element transfer from root to shoot. These findings were consistent with prior studies on watermelon, lettuce, coriander, and cucumber (Matraszek et al., 2017; Nawaz et al., 2018; Cao et al., 2019; Sardar et al., 2022b).

## Conclusion

**FIGURE 9:**
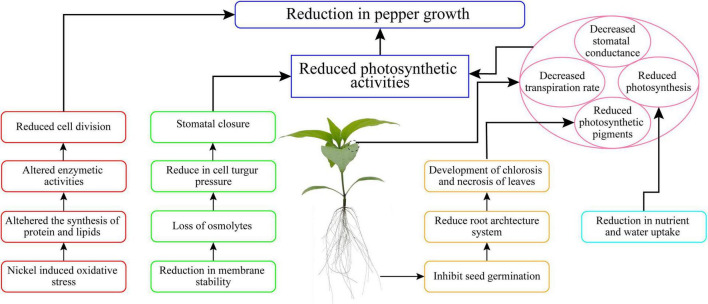
Summary of the Ni stress tolerance mechanism in pepper seedlings.

In the current work, we demonstrated that Ni stress led to significant growth retardation, limitations of the photosynthetic apparatus, mineral homeostasis imbalances, and the formation of excessive ROS, and all of these collectively reduced Ni-stress tolerance in pepper seedlings (Figure 9). In this investigation, increased Ni concentrations (10, 20, 30, 50, 75, and 100 mg L^–1^ Ni) inhibited the pepper seedling growth by affecting the physiological, morphological, and biochemical characteristics. The findings confirmed that Ni at 50, 75, and 100 mg L^–1^ considerably decreased pigment content and leaf gas exchange elements, resulting in a reduction in seedling growth, but V at 10, 20, and 30 mg L^–1^ produced a less significant reduction in seedling growth. The application of Ni stunted root development, and it was observed that Ni was more accumulated in the roots than in the shoot of pepper seedlings. The results of this research also suggest that higher Ni (50, 75, and 100 mg L^–1^) concentration may play a negative role in the cells of pepper plants. The cell death in pepper plants happened owing to oxidative damage induced by Ni, leading to the formation of ROS which enhanced the antioxidant enzyme activity. Further study is required in the future to investigate the mechanism and gene expression involved in cell death induced by Ni toxicity in pepper plants.

## Data Availability Statement

All data generated or analyzed during this study are included in this article.

## Author Contributions

MA: conceptualization, methodology, and writing—original draft. CH, YH, and HS: investigation. MAM and HF: formal analysis. MAM and ZW: review and editing. ZW: project administration, resources, and supervision. All authors contributed to the article and approved the submitted version.

## Conflict of Interest

The authors declare that the research was conducted in the absence of any commercial or financial relationships that could be construed as a potential conflict of interest.

## Publisher’s Note

All claims expressed in this article are solely those of the authors and do not necessarily represent those of their affiliated organizations, or those of the publisher, the editors and the reviewers. Any product that may be evaluated in this article, or claim that may be made by its manufacturer, is not guaranteed or endorsed by the publisher.
